# Relationships between Pain, Function and Radiographic Findings in Osteoarthritis of the Knee: A Cross-Sectional Study

**DOI:** 10.1155/2012/984060

**Published:** 2012-11-19

**Authors:** Duygu Cubukcu, Ayse Sarsan, Hakan Alkan

**Affiliations:** ^1^Department of Physical Medicine and Rehabilitation, Dr. Behcet Uz Children's Diseases Training and Research Hospital, Sezer Dogan Street No. 11, Konak, 35210 Izmir, Turkey; ^2^Department of Physical Medicine and Rehabilitation, Pamukkale University Faculty of Medicine, Kınıklı, 20070 Denizli, Turkey

## Abstract

*Objectives*. The aim of this study was to investigate the relationships between pain, disability, and radiographic findings in patients with knee osteoarthritis (OA). *Patients and Methods*. A total of 114 patients with knee OA who attended the physical medicine and rehabilitation outpatient clinic were included in this study. The diagnosis was based on the American College of Rheumatology (ACR) criteria for knee OA. Age, duration of disease, and body mass index (BMI) of the patients were recorded. Radiographic features on the two-sided knee radiography were assessed with the Kellgren-Lawrence scale. The severity of knee pain, stiffness, and disability were measured using the Western Ontario and McMaster Universities Osteoarthritis Index (WOMAC). *Results*. The mean age of the patients was 56.98 (±8.28) years and the mean disease duration was 4.14 (±4.15) years. Kellgren-Lawrence grading scale and age or disease duration were positively and significantly associated, whereas none of the WOMAC subscores were found to be related with Kellgren-Lawrence grading scale (*P* > 0.05). On the other hand, WOMAC disability scores were significantly associated with WOMAC pain and WOMAC stiffness (*P* < 0.01). *Conclusions*. Knee pain, stiffness, and duration of disease may affect the level of disability in the patients with knee OA. Therefore treatment of knee OA could be planned according to the clinical features and functional status instead of radiological findings.

## 1. Introduction

Osteoarthritis (OA) is the most common degenerative joint disorder and a major public health problem throughout the world. It affects any joint containing hyaline cartilage [1, 2] and the knees are the most commonly affected joints [3]. Prevalence of OA increases with age and aging is associated with decreasing physiological functions, thus leading to major health problems. As a larger proportion of the elderly population in developed countries increasingly lives to an extreme old age, OA will be more prevalent and will be an important cause of disability in the future [4]. 

Various treatment strategies are recommended, which are aimed to reduce symptoms and prevent further functional deterioration [5, 6]. While planning rehabilitation or making arthroplasty decision many physicians take into consideration the radiographic features. It is important that we have a clear understanding about the relationship between function and radiographic features. In this study, we aimed to determine if there was a positive correlation between pain, disability, and radiographic findings in patients with knee OA. 

## 2. Patients and Methods

In this paper, we describe a cross-sectional analysis designed specifically to assess the relationships between pain, function, and radiographic features of knee OA. One hundred and sixty-five patients who attended the Physical Medicine and Rehabilitation Clinic at the Pamukkale University between September 2006 and April 2007 for knee pain for at least one year and were diagnosed as having knee OA according to the American College of Rheumatology (ACR) Classification Criteria [7] were included in this study. Exclusion criteria were inflammatory knee disorders, other arthropathies, metabolic bone disease, serious systemic diseases, depression, neoplasms, history of knee trauma or knee surgery, and previous intra-articular injections. After demographic information and disease duration were recorded, detailed physical examination including anthropometric measures for determination of body mass index (BMI) was performed. 

All patients were informed about the aims of the study and the study protocol, and their informed consents were obtained prior to the study.

**Disability and Functional Status**

The physical function section of the Western Ontario and McMaster Universities Osteoarthritis Index (WOMAC) was used as an indicator of self-reported disability [8]. This section of the WOMAC evaluates 17 activities (WOMAC C). Subjects rated the degree of difficulty experienced in the preceding 48 hours for each of the 17 activities using a 5-level numeric verbal descriptor scale with a total subcore ranging from 0 to 68. The activities included in the WOMAC physical function section occur commonly on a daily basis and have faced validity for lower limb function. Higher scores indicate greater levels of difficulty.

**Pain**

Pain was assessed using the pain section of the WOMAC (WOMAC A) [8]. This measure of pain includes 5 summed items and is commonly used as an indicator of OA knee pain. Total subscore for pain can range between 0 and 20.

**Stiffness**

Stiffness was assessed using the stiffness subscale of the WOMAC (WOMAC B) which includes two items and has a total subcore of zero to eight [8].

**Radiography**

Weight-bearing anteroposterior and lateral semiflexed radiographs were recorded for both knees in each subject. They were radiologically graded according to the Kellgren-Lawrence Index [9]. The Kellgren-Lawrence grading scale is a reliable and valid testing tool used in conjunction with radiograph. This method is widely used in the diagnosis as well as in epidemiologic studies on OA of the knee and was accepted by the World Health Organization [10]. The Kellgren-Lawrence scoring used ratings from 0 to 4, where 0 = normal radiograph; 1 = doubtful pathology; 2 = minimal osteophytes, possible narrowing, cysts, and sclerosis; 3 = moderate, as in definite osteophytes with moderate joint space narrowing; 4 = severe, with large osteophytes and definite joint space narrowing. Each radiograph was evaluated by an experienced observer who was blinded to patients' details.

**Statistical Analysis**

All statistical analyses were performed using SPSS version 12 for Windows. Descriptive statistics were used to describe demographic characteristics. Spearman's rank correlation coefficients were calculated to determine the relationships between clinical parameters and radiographic grades in patients with knee OA. Kruskal-Wallis test was used to analyze if there were any significant differences in the level of pain, disability and stiffness according to Kellgren-Lawrence grading scale. In all analyses, *P* values <0.05 were considered statistically significant.

## 3. Results

**Figure 1:**
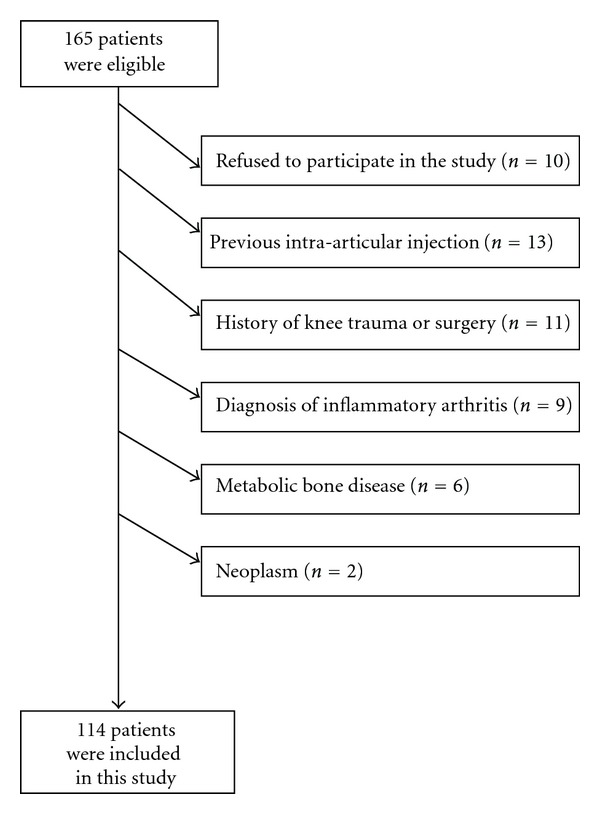
Flowchart of the study.

One hundred and sixty-five community-based patients who attended the research clinic with OA in at least one of their knees were invited to participate in the study. Fifty-one patients were excluded from the study for some reasons as shown in flowchart of the study (Figure 1). One hundred and fourteen patients with knee OA who were eligible for the current analysis were included in this study. The ages of the OA patients were between 40 and 74 years (mean 56.98 ± 8.28) and the majority of the OA patients was females. The disease duration of knee OA patients was between 1 and 20 (mean 4.14 ± 4.15) years. Seventy-three percent of the patients reported bilateral knee pain. The mean BMI score was 29.15 ± 4.39 kg/m².

On the radiographic assessment, 12 patients (10.5%) were grade 1 on the Kellgren-Lawrence Index, 39 (34.2%) were grade 2, 57 (50.0%) were grade 3, and 6 (5.3%) had grade 4, showing that the subjects were mostly categorized as mild to moderate for radiographic features. The demographic, clinical, and radiological data of the patients are presented in Table 1.

Kellgren-Lawrence grading scale and age or disease duration were positively and significantly associated (*P* < 0.05). Whereas none of the WOMAC subscores were found to be related with Kellgren-Lawrence grading scale (*P* > 0.05). On the other hand, WOMAC disability scores were significantly associated with WOMAC pain and WOMAC stiffness (*P* < 0.01). The correlations between clinical variables, WOMAC subscores, and Kellgren-Lawrence scores are summarized in Table 2. Also there was no statistically significant difference in the level of pain, disability, and stiffness according to Kellgren-Lawrence grading scale as shown in Table 3.

## 4. Discussion

In this cross-sectional study we investigated if there was any association between pain, disability, and radiographic features in patients with knee OA. Our results demonstrated that age and disease duration were found to be positively associated with Kellgren-Lawrence grading scale. Also disability scores were significantly associated with pain and stiffness scores as measured by WOMAC. However, we could not establish an association between Kellgren-Lawrence grading scale and WOMAC subscores. 

Knee OA is particularly important in view of its high prevalence and association with severe pain and disability [11]. Pain is the main complaint among patients with knee OA, a leading cause of physical disability [12]. The risk of disability increases with the presence of knee pain in the community [13–15]. Thus it is important to understand the factors which contribute to disability in patients with knee OA. There are some studies which report the relationship between pain and physical functions in patients with knee OA [16–20]. McAlindon et al. [16] demonstrated that knee pain and age are more important determinants of functional impairments in elderly subjects than the severity of knee OA as assessed by radiographic features. In another study it was reported that the disability index was related to the severity of pain assessed either through the McGill Pain Questionnaire or a visual analog scale [17]. Also, Jordan et al. concluded that knee pain severity was more important than radiographic knee OA severity in determining disability [18]. In addition Creamer et al. concluded that the function in knee OA is determined more by pain and obesity than by structural changes seen on plain radiographs [19]. In accordance with these studies, we demonstrated a positive correlation between pain severity and disability when assessed by the WOMAC subscales. The WOMAC scale allows a detailed analysis of pain. Using the WOMAC pain subscale, patients score the pain severity while performing specific activities. We considered this finding as a result of vicious consisting pain leading to decreased functional ability. 

It is important that we have a clear understanding about the relationship between function and radiographic features. However, few studies [16, 19, 21] attempted to assess the relationship between radiographic features and function in patients with knee OA. Larsson et al. [21] reported that radiographic diagnosis of osteoarthritis was not related to functional capacity. Also in some studies, correlation between self-reported disability and radiographic change could not be established [16, 19]. Similar to these findings we found no correlation between function and radiographic features. In contrast to these findings, it was demonstrated that knee pain and reduced function were more likely to be found if radiographic OA features were present in both tibiofemoral (medial and/or lateral) and also patellofemoral compartments rather than the involvement of only either of them [22].

The clinical parameters and radiographic findings were both important in the diagnosis and management of OA. Diagnosis of OA, according to radiological findings without clinical signs of disease, leads to unnecessary drug use in the elderly. Thus it is important to determine the relationships between clinical variables and radiographic findings. A survey of the literature has shown disagreements between clinical variables and radiological investigations [1, 15, 23–25]. In recent studies, it has been shown that the Kellgren-Lawrence score was not related to WOMAC score but that it was important to follow up the progress of the disease [26, 27]. The Framingham Osteoarthritis Study found that 10% of people aged 63 years and over had symptomatic knee OA in the presence of radiographic changes [1]. Individuals with radiographic evidence of OA may be asymptomatic at any time. In the relevant literature, results have been conflicting as some studies [17, 28] reported no association between pain scores and radiographic features and others [16, 25, 29] found that radiographic features of OA were significantly associated with knee pain. In this study, radiological findings did not correlate with the severity of pain as assessed by WOMAC. These results may be due to our patients' characteristics since they were mostly categorized as mild to moderate for radiographic features in our study. It is possible that pain bears a stronger relationship to radiographic features in patient with severe disease. On the other hand, conventional radiography which is the most commonly used imaging modality may not identify bony changes related to pain in early knee OA. Radiographs demonstrate structural changes rather than disease severity. Conventional radiography permits only limited assessment of the three knee compartments, provides only an approximation of articular cartilage change with measurement of joint space narrowing, and poorly characterizes other soft tissues [30].

Secondary changes occurring in the joint with increasing age cause OA to be one of the major health problems in the elderly. In the epidemiological studies, the relationship between age and OA was found to be the most striking finding [11, 14]. In a recent study it was demonstrated that radiographic findings were found to be related with age and disease duration which shows the progressive nature of OA [26]. We also found a positive correlation between age, disease duration, and radiographic findings. Increased prevalence of OA with advanced age may be due to changes in cartilage with aging, muscle weakness, the loss of chondrocytes, the loss of flexibility of subchondral bone, and inadequate neuromuscular response facilitating joint damage. 

Symptomatic knee osteoarthritis in women has been reported more frequently than that in men [19, 31, 32]. In our study, the number of female patients with knee OA is striking. This condition could be partly explained in aging women with the differentiation of the hormonal status and the imbalance in the formation and destruction of bone. Menopause has been associated with an increased production of interleukin-1 which is the part of the cytokine response in OA. In postmenopausal women as the level of estrogen decreases interleukin-1 levels can increase which leads to OA [33].

Potential limitations of this study are its cross-sectional design rather than longitudinal followup and relatively small number of participants who had severe disease as assessed by radiographic features. Moreover we used only composite scores of joint damage that do not adequately reflect bone changes rather than individual radiographic features. In addition age, gender, and the WOMAC scale were the patient-related data we collected, so we were not able to assess the separate contribution of possible confounders that have been associated with pain, disability, and function in knee OA, such as comorbidities which reduced the statistical power of our study.

## 5. Conclusions

We conclude that knee pain, stiffness, and duration of disease may affect the level of disability in the patients with knee OA. Therefore it would be better to consider mainly the functional status of patients in addition to clinic and radiological findings while planning the treatment of OA. Further longitudinal research would identify the rate of radiographic progression and its associations with change in pain and function.

## Figures and Tables

**Table 1:** Demographic features, clinical, and radiological characteristics of the patients.

Age (years) (mean ± SD)	56.98 ± 8.28
BMI (kg/m^2^) (mean ± SD)	29.15 ± 4.39
Gender	
Female *n* (%)	99 (86.8)
Male *n* (%)	15 (13.2)
Disease duration (years) (mean ± SD)	4.14 ± 4.15
Kellgren-Lawrence scale, *n* (%)	
Grade I	12 (10.5)
Grade II	39 (34.2)
Grade III	57 (50.0)
Grade IV	6 (5.3)
WOMAC pain score (mean ± SD)	14.42 ± 3.35
WOMAC stiffness score (mean ± SD)	5.52 ± 1.86
WOMAC function score (mean ± SD)	46.92 ± 9.04

WOMAC: Western Ontario and McMaster Universities Osteoarthritis Index.

BMI: Body mass index.

**Table 2:** Correlation coefficients showing associations between radiographic grade and clinical parameters.

	BMI (r)	Disease duration (r)	WOMAC-A (r)	WOMAC-B (r)	WOMAC-C (r)	Kellgren-Lawrence (r)
Age	−0.223*	0.217*	0.081	−0.049	0.114	0.367**
BMI		0.173	0.10	0.037	0.169	0.149
Disease duration			0.102	0.151	0.136	0.386*
WOMAC-A				0.342**	0.631**	−0.046
WOMAC-B					0.331**	−0.119
WOMAC-C						0.172

*P < 0.05 and **P < 0.01.

WOMAC: Western Ontario and McMaster Universities Osteoarthritis Index.

BMI: Body mass index.

**Table 3:** Level of pain, disability, and stiffness according to the four radiographic stages.

	KL Grade I (mean ± SD)	KL Grade II (mean ± SD)	KL Grade III (mean ± SD)	KL Grade IV (mean ± SD)	*P*
WOMAC-A	16,42 ± 2,50	14,00 ± 3,49	13,98 ± 3,34	16,86 ± 2,27	>0.05
WOMAC-B	6,92 ± 2,07	5,41 ± 1,94	5,19 ± 1,69	6,57 ± 1,13	>0.05
WOMAC-C	48,67 ± 7,04	44,02 ± 9,62	47,86 ± 8,55	53,00 ± 9,25	>0.05

KL: Kellgren-Lawrence scale.

WOMAC: Western Ontario and McMaster Universities Osteoarthritis Index.

## References

[1] Felson DT, Naimark A, Anderson J (1987). The prevalence of knee osteoarthritis in the elderly. The Framingham Osteoarthritis Study. *Arthritis and Rheumatism*.

[2] Puett DW, Griffin MR (1994). Published trials of nonmedicinal and noninvasive therapies for hip and knee osteoarthritis. *Annals of Internal Medicine*.

[3] Davis MA (1988). Epidemiology of osteoarthritis. *Clinics in Geriatric Medicine*.

[4] Hammerman D (1995). Clinical implications of osteoarthritis and ageing. *Annals of the Rheumatic Diseases*.

[5] Smidt N, de Vet HC, Bouter LM (2005). Effectiveness of exercise therapy: a best-evidence summary of systematic reviews. *Aust J Physiother*.

[6] Bellamy N, Campbell J, Robinson V, Gee T, Bourne R, Wells G (2005). Viscosupplementation for the treatment of osteoarthritis of the knee. *Cochrane Database of Systematic Reviews*.

[7] Altman RD (1991). Criteria for classification of clinical osteoarthritis. *Journal of Rheumatology*.

[8] Bellamy N, Buchanan WW, Goldsmith CH, Campbell J, Stitt LW (1988). Validation study of WOMAC: a health status instrument for measuring clinically important patient relevant outcomes to antirheumatic drug therapy in patients with osteoarthritis of the hip or knee. *Journal of Rheumatology*.

[9] Kellgren HJ, Lawrence SJ (1967). Radiological assessment of osteoarthritis. *Annals of the Rheumatic Diseases*.

[10] WHO Scientific Group (1992). Rheumatic diseases.

[11] Felson DT, Brandt KD, Doherty M, Lohmander LS (2003). Epidemiology of osteoarthritis. *Osteoarthritis*.

[12] Torres L, Dunlop DD, Peterfy C (2006). The relationship between specific tissue lesions and pain severity in persons with knee osteoarthritis. *Osteoarthritis and Cartilage*.

[13] Guccione AA, Felson DT, Anderson JJ (1990). Defining arthritis and measuring functional status in elders: methodological issues in the study of disease and physical disability. *American Journal of Public Health*.

[14] Davis MA, Ettinger WH, Neuhaus JM, Mallon KP (1991). Knee osteoarthritis and physical functioning: evidence from the NHANES I epidemiologic followup study. *Journal of Rheumatology*.

[15] Davis MA, Ettinger WH, Neuhaus JM, Barclay JD, Segal MR (1992). Correlates of knee pain among US adults with and without radiographic knee osteoarthritis. *Journal of Rheumatology*.

[16] McAlindon TE, Cooper C, Kirwan JR, Dieppe PA (1993). Determinants of disability in osteoarthritis of the knee. *Annals of the Rheumatic Diseases*.

[17] Falaffi F, Cavalieri F, Nolli M, Ferraccioli G (1991). Analysis of disability in knee ostoarthritis. Relationship with age and psychological variables but not with radiographic score. *Journal of Rheumatology*.

[18] Jordan JM, Luta G, Renner JB (1996). Self-reported functional status in osteoarthritis of the knee in a rural southern community: the role of sociodemographic factors, obesity, and knee pain. *Arthritis Care and Research*.

[19] Creamer P, Lethbridge-Cejku M, Hochberg MC (2000). Factors associated with functional impairment in symptomatic knee osteoarthritis. *Rheumatology*.

[20] Ay S, Evcik D (2008). Effectiveness of pain, disease severity and radiological grading on disability of daily living activities in knee osteoarthritis. *Romatizma*.

[21] Larsson AC, Petersson I, Ekdahl C (1998). Functional capacity and early radiographic osteoarthritis in middle-aged people with chronic knee pain. *Physiotherapy Research International*.

[22] Szebenyi B, Hollander AP, Dieppe P (2006). Associations between pain, function, and radiographic features in osteoarthritis of the knee. *Arthritis and Rheumatism*.

[23] Kellgren JH, Lawrence JS (1952). Rheumatism in miners. II. X-ray study. *British Journal of Industrial Medicine*.

[24] Lawrence JS, Bremner JM, Bier F (1966). Osteo-arthrosis. Prevalence in the population and relationship between symptoms and x-ray changes. *Annals of the Rheumatic Diseases*.

[25] Lethbridge-Cejku M, Scott WW, Reichle R (1995). Association of radiographic features of osteoarthritis of the knee with knee pain: data from the Baltimore Longitudinal Study of Aging. *Arthritis Care and Research*.

[26] Külcü DG, Yanık B, Atalar H, Gülsen G (2010). Associated factors with pain and disability in patients with knee osteoarthritis. *Turkish Journal of Rheumatology*.

[27] Rupprecht TN, Oczipka F, Lüring C, Pennekamp PH, Grifka J (2007). Is there a correlation between the clinical, radiological and intrasurgical findings of osteoarthritis of the knee? A prospective study on 103 patients. *Zeitschrift fur Orthopadie und Unfallchirurgie*.

[28] Hannan MT, Felson DT, Pincus T (2000). Analysis of the discordance between radiographic changes and knee pain in osteoarthritis of the knee. *Journal of Rheumatology*.

[29] Cicuttini FM, Baker J, Hart DJ, Spector TD (1996). Association of pain with radiological changes in different compartments and views of the knee joint. *Osteoarthritis and Cartilage*.

[30] Hodler J, Resnick D (1996). Current status of imaging of articular cartilage. *Skeletal Radiology*.

[31] Brandt KD, Fife RS (1986). Ageing in relation to the pathogenesis of osteoarthritis. *Clinics in Rheumatic Diseases*.

[32] Cushnaghan J, Dieppe P (1991). Study of 500 patients with limb joint osteoarthritis. I. Analysis by age, sex, and distribution of symptomatic joint sites. *Annals of the Rheumatic Diseases*.

[33] Sowers M (2001). Epidemiology of risk factors for osteoarthritis: systemic factors. *Current Opinion in Rheumatology*.

